# The Relevance of Explanatory First-Person Approaches (EFPA) for Understanding Psychopathological Phenomena. The Role of Phenomenology

**DOI:** 10.3389/fpsyg.2018.00694

**Published:** 2018-06-20

**Authors:** Philipp Schmidt

**Affiliations:** ^1^Department of Philosophy, University of Vienna, Vienna, Austria; ^2^Phenomenological Psychopathology and Psychiatry, University Clinic Heidelberg, Heidelberg, Germany

**Keywords:** first-person experience, explanation, phenomenology, introspection, Cognitive Theory, depression, temporality, embodiment

## Abstract

The main aim of this paper is to demonstrate the contributions of phenomenology-inspired approaches to the explanation of psychopathological phenomena. First, I introduce the notion of Explanatory First-Person Approaches (EFPA) which share the assumption that the explanation of consciousness and conscious phenomena necessitates, at least partially, phenomenal facts functioning as explanans. Phenomenal facts refer to facts about structures and processes of consciousness. To differentiate phenomenology from other EFPA and to extract its distinctive feature, I compare phenomenology to the method falling under the category of EFPA it overlaps with the most: new introspective methods as recently described. I then present genetic phenomenology as the distinctive feature of phenomenology and show how particularly genetic phenomenology complements biological explanations of psychopathological phenomena in the context of psychiatric disorders such as schizophrenia. Moreover, I present Cognitive Theory (CT) as the most acknowledged EFPA in the broader scientific community, demonstrate CT’s limitations in explaining conscious phenomena in the context of psychological disturbances such as depression, and show how genetic phenomenology can also significantly complement the cognitive approach. An example in the context of burnout-depression will be given. The overall argument for the significance of phenomenology is as follows: Genetic phenomenology renders phenomenology a distinctive kind of EFPA; genetic phenomenology can complement one of the most dominant non-EFPA accounts in the science of psychiatric disorders: biological reductionism; and genetic phenomenology can complement the most dominant existing EFPA in the science of psychological disturbances: Cognitive Theory.

## Introduction

Consciousness remains one of the most controversial issues in philosophy. Its study brings together numerous disciplines such as psychology, psychiatry, sociology, and the cognitive sciences more generally. Important topics of recent debate concern not only the concept and structure of consciousness. The question as to how the study of consciousness is to inform philosophical investigations of more general kind, related to widely varying philosophical issues such as knowledge or personal identity, is much debated too. Moreover, despite the long tradition of consciousness research, methodological questions as to how one is to perform an analysis of consciousness and how to assess the explanatory power of the methods proposed are also still being discussed widely.

Addressing the explanatory power of one family of consciousness research approaches, the main aim of this paper is to demonstrate the contributions phenomenology-inspired accounts can make to the explanation of psychopathological phenomena. In Section “The Relationship between Introspection and Phenomenology, and the Concept of Explanatory First-Person Approaches (EFPA),” I discuss the relationship between contemporaneous introspective approaches and phenomenology. I argue that phenomenology presents its own kind of analysis, although phenomenology and introspection share the idea that phenomena of consciousness are best explained by the structures of consciousness themselves. Moreover, I suggest that this idea is also shared by other methods and that the term *Explanatory First-Person Approaches* (EFPA) covers this notion in an informative and robust way. In Section “Phenomenological Psychopathology and Explanation: Genetic-phenomenological Aspects of Experience,” I describe genetic phenomenology as a unique feature of phenomenology that distinguishes it from other EFPA. Moreover, I reconstruct why genetic phenomenology has been unpopular in the tradition of phenomenological psychopathology. I then go on to show how genetic analyses can inform explanations in psychiatric disorders. In Section “Phenomenology and the Cognitive Approach: The Explanatory Role of Phenomenology in Psychological Disturbances,” I turn to a different EFPA which has become the mainstream exponent of EFPA in the broader scientific community: Cognitive Theory (CT). I argue that CT shows serious limitations in doing justice to the complexity of phenomenal experience and that genetic phenomenology is apt to complement CT in explaining psychological disorders, e.g., burnout and depression.

## The Relationship Between Introspection and Phenomenology, and the Concept of Explanatory First-Person Approaches

While introspection was the standard method in the young discipline of psychology used by protagonists such as Wundt, Brentano, Titchener, Külpe, or Bühler, it was superseded by behaviorism. Even though phenomenal consciousness regained great importance as a subject of psychology through the growing influence of cognitivism later into the century, introspection never fully recovered its reputation. This is not to say that it ceased to exist. Quite the contrary, in the last two decades, introspective methods have been further developed and brought into the field as new and more fine-grained approaches. Unlike traditional approaches, which focused on the introspection of experiential changes induced by stimuli and carried out by well-trained psychologists under experimental conditions, new introspective methods (NIM) direct their attention to all kinds of situations as they are lived through. This includes the layperson. Moreover, NIM have lived experience as a whole as their subject of analysis, rather than the experiential impact of a single stimulus or group of stimuli. Trying to carve out what it is like to undergo a certain experience *x*, their investigation focuses on tacit aspects of *x* and how they render *x* the kind of experience it is. Examples of NIM are the Descriptive Experience Sampling (DES; Hurlburt and Akhter, 2006; Hurlburt, 2011) or the Explicitation Interview (EI; Petitmengin, 2006; Maurel, 2009; Petitmengin and Bitbol, 2009; Vermersch, 2009).

Although Husserl and other phenomenologists have emphasized the difference between introspection and phenomenology (Merleau-Ponty, 1945, p. 70; Gurwitsch, 1966, pp. 89–106; Heidegger, 1993, pp. 11–17), interestingly, some proponents of NIM have sought an alliance with the phenomenological tradition (Vermersch, 2009, p. 25).

### The Overlap Between New Introspective Methods and Phenomenology

Zahavi (2011, p. 17 f.) has responded negatively to this attempt at approximation, stressing that not only the goals of phenomenology differ from introspective psychology but also the means to achieve them. Most importantly, phenomenology is neither interested in psychological processes nor “concerned with establishing what a given individual might currently be experiencing” (Zahavi, 2011, p. 18). Rather, phenomenology is a philosophical analysis of the world and different kinds of appearances, their specific way of givenness and their “essential structures and conditions of possibility” (Zahavi, 2011, p. 18). To do so, phenomenology does not rely on “turning our gaze inward (introspicio),” but is “paying attention to how worldly objects and states of affairs appear to us” (Zahavi, 2011, p. 17).

All of this is certainly true, particularly for classical phenomenology. However, there are at least three reasons why these remarks do not sufficiently distinguish phenomenology from NIM.

First, it is not clear to what extent the new introspectionists insist on having their approach called “introspection.” Some proponents came to problematize the term (Petitmengin and Bitbol, 2009, p. 379), but defend “introspection from within” (Bitbol and Petitmengin, 2013, p. 269) in a more recent article. However, despite using the term “introspection,” they intend to conceptualize it in terms of an “‘enlargement of the field and contact with re-enacted experience,’ rather than ‘looking-within”’ (Bitbol and Petitmengin, 2013, p. 269). Moreover, in a reply to Zahavi, Vermersch admits that “introspection” is a metaphor, but maintains that the alternatives proposed by Husserl “constitute no improvement” as they all relate to “the metaphor of ‘view’ or ‘spection”’ (Vermersch, 2011, p. 20–21; Zahavi, 2011).

Second, as Sass and Fishman (forthcoming) elaborate, some of the conceptual and methodological aspects developed by and specific to phenomenology can be found, though in a somewhat different way, in NIM. Sass and Fishman (forthcoming) give the following examples: the bracketing of theoretical assumptions concerning consciousness corresponding to the phenomenological concept of *epoché*; the attentional shift “from the narrow *content* to the complete *act* of consciousness” corresponding to *phenomenological reduction* (Bitbol and Petitmengin, 2013, p. 273); and the emphasis on the mitigation of the traditional distinction between the internal, the mind, and the external, the world (Petitmengin and Bitbol, 2009, p. 379), corresponding to the basic structure of intentionality essential to conscious existence. Moreover, given that the interviewer in EI assists the interviewee in focusing on and making explicit implicit or tacit aspects of their lived experience, Sass and Fishman (forthcoming) conclude that the explicitationists’ method embraces three constitutive aspects of phenomenological methodology: epoché, descriptive analysis and intersubjective validation (cf. Gallagher and Zahavi, 2012).

A third reason why Zahavi’s rather narrow delineation of phenomenology is not entirely convincing refers to the central and transcendental goal of phenomenology: the carving out of the essentials of the appearance of different kinds of being and their constitution in phenomenal consciousness. Not only is there considerable variety within phenomenological philosophy, there is also a well-established practice of phenomenological psychopathology that, despite working with transcendental insights into the general character of consciousness and contributing to the science of the latter, is also concerned with specific psychological processes constitutive of types of abnormal experience (e.g., Jaspers, 1973; Fuchs, 2001, 2003, 2013a,b, 2014; Sass and Parnas, 2007; Ratcliffe, 2008, 2015; Stanghellini et al., 2016). To this end and to gain a better understanding of a certain psychological condition, phenomenological psychopathology is therefore indeed sometimes interested in what a “given individual might currently be experiencing” (Zahavi, 2011, p. 18). For, as Sass and Fishman (forthcoming) stress, illuminating psychopathological phenomena is something that needs more than a purely eidetic form of intuition. It requires complementation by an adapted sort of phenomenological analysis from a second or third-person perspective (Sass and Fishman, forthcoming; cf. Ratcliffe, 2015, pp. 16–32).

Given these aspects, the relationship between phenomenology and NIM, let alone whether the latter should be denominated ‘introspection’ at all, remains unclear. In what follows, I will suggest a different term under the heading of which one might group NIM and phenomenology bearing in mind their overlap as well as their differences.

### The Idea of Explanatory First-Person Approaches

Zahavi’s rejection of the affinity between phenomenology and NIM has proven to be too strict. However, it is indeed fair to say that whatever features phenomenology and NIM have in common, they are far from being exactly the same. To have claimed this, was, as Vermersch acknowledges, a “provocation” (Vermersch, 2011, p. 20). But this confession does not seem to prevent him from continuing to maintain that phenomenology and NIM – regardless of whether one calls it introspection or otherwise – perform the very same act. And it is here, when he points to the “varieties of reflection,” that Zahavi (2011) is right. I take this to be the important message of his rejection of introspection, which is too strong considering the *similarity* between phenomenology and NIM on the one hand, but adequate when considering the claim of *equation* of phenomenological reflection with introspection on the other hand. After all, if Vermersch is right that “the real problem is still to determine the nature of this act whether we call it introspection or reflection or whatever” (Vermersch, 2011, p. 22), then it does not make sense to maintain the sheer identity of both cases. For if the nature of the allegedly same act is yet to be revealed, how could one possibly make the judgment that phenomenological reflection and introspection refer to the very same act?

Therefore, any account of the relationship between phenomenology and introspective methods should start with the assumption of the plurality of ways in which a subject can attend to and describe its lived experience. Moreover, it should take into consideration that descriptive data can be analyzed in different ways. Once the nature of the acts – each method in its full scope – is completely specified, one can then test them for congruency. Having said this, even without a complete determination of the methods in question, it is possible – as Sass and Fishman (forthcoming) demonstrate – to acknowledge a high degree of affinity between phenomenology and NIM.

But is this grouping of phenomenology and NIM informative in any interesting way? If the main reason to consider them as members of the same theory-family is merely that they rely on a kind of description of first-personal experience, then the grouping is not far from being irrelevant, because even naturalistic and reductionist accounts of consciousness require a certain degree of phenomenological reflection, introspection of processes of the inner sense, or descriptions of first-personal experience: they need to define their explananda – in the most accurate way.

But then phenomenology and NIM fall under the same category together with other approaches both seek to distinguish themselves from. Any meaningful classification, therefore, must go beyond the feature of making some reference or other to first-personal experience. One might then bring in the features Sass and Fishman (forthcoming) have pointed out as similarities – such as bracketing theoretical assumptions or the aim to reveal tacit aspects of the full act of consciousness.

However, there is another issue more apt to group phenomenology and NIM, one that distinguishes them from reductionist accounts. While the latter accept and are dependent on first-personal experience as their explananda, both phenomenology and NIM invest their descriptive effort in not only providing an accurate definition of their explanandum. Rather, describing and analyzing lived experience, they intend to give *explanations* of different kinds of experiences *in terms* of first-personal experience. In other words, they take first-personal experience not only to be the explanandum but also to function as explanans. This means that in their attempts to carve out experiential structures, their components and processes, they intend to give explanations of what makes a type (phenomenology) or token (EI) of experience the specific experience it is.

Based on this criterion there is a meaningful way to distinguish between approaches that refer differently to first-personal experience: (1) those that describe and analyze first-personal experience as explananda to be explained by non-experiential or non-phenomenological mechanisms such as underlying neurobiological processes or socio-economic factors etc. and (2) those that explain first-personal experience by revealing their structure, relevant tacit aspects and processes, and their experiential unfolding over time.

Given the high heterogeneity within (1) compared to the more homogenous variety of approaches within (2), I will only propose a denomination for (2). I will suggest referring to (2) as *Explanatory First-Person Approaches*.

### Five Reasons Why the Term EFPA Is a Good Choice

The reasons for this rather general notion are as follows. First, the main criterion for accounts to fall in this category is the assumption that processes and structures of first-personal experience and the analysis thereof have explanatory power regarding psychological phenomena. EFPA analyze first-personal experience to understand and explain first-personal experiences.

Second, there are many ways of performing such an analysis. Some approaches might overlap with phenomenology and/or introspective methods to some degree, but also make use of other methods to gain insight into the lived experience of persons and its structure. A prominent example is Cognitive Theory (CT) (Beck et al., 1979; Beck and Haigh, 2014). Their founders explicitly mention “Kant, Heidegger, and Husserl” and their “philosophical emphasis on conscious subjective experience” as having “substantially influenced the development of modern psychology in this group of psychotherapies [i.e., Cognitive Therapy]” (Beck et al., 1979, p. 9). They also acknowledge the assumption that “perception and experiencing in general (…) involve both inspective and introspective data” (Beck et al., 1979, p. 8). However, their method to carve out a person’s “way in which he structures the world” by which “an individual’s affect and behavior are largely determined” (Beck et al., 1979, p. 3) is distinctive in kind. Their focus lies on identifying pathogenic beliefs and thinking schemas that are said to underlie lived experience as tacit aspects. This identification is to be achieved by personal interlocution or written interview techniques. Despite their overlap with phenomenology and introspective methods, they present a sui generis approach which nonetheless takes the analysis of the structure of first-personal experiences to have explanatory power. For this reason alone, neither “phenomenology” nor “introspection” is suitable for the title of (2).

Third, other possible alternatives such as “reflective approaches” or “descriptive accounts” do not cover the full spectrum of accounts that emphasize the explanatory power of the analysis of experiential structures. For, not all approaches and involved methods consist in merely self-aware reflection on one’s own experiences. Another possibility, “psychological accounts,” is misleading and perpetuates the traditionally problematic strict distinction between ‘psyche’ and ‘soma.’

Fourth, to count as an EFPA, no commitment to a specific understanding of how a subject is aware of herself and her own experiences is required. The notion of EFPA allows for a wide range of different approaches concerning the exact nature of self-awareness, introspection and self-knowledge, which is subject to historic and on-going controversy (e.g., Zahavi, 1999; Kriegel and Williford, 2006; Gertler, 2011; Strawson, 2017). This means the term EFPA is not only open to a variety of methods applied to obtain first-personal experiential data, but also to a variety of theories concerning the nature of consciousness and specific acts of consciousness (cf. Feest, 2012, 2014).

Fifth, EFPA is, however, just robust and specific enough to distinguish approaches in question from all other accounts that address first-personal experience in one way or another and that are frequently referred to in more general terms such as “first-person methods” (Feest, 2014) or “first-person approaches” (Varela and Shear, 1999).

To conclude this section, my proposal is to consider phenomenology and NIM as members of a broader group of approaches. While approaches falling under this label share the common feature of taking the inquiry into first-personal experience to have an explanatory function, they may vary in both their method of analyzing first-personal experience and how they conceptualize first-personal experience. Given this heterogeneity, each sub-type of EFPA should be evaluated individually concerning its explanatory and overall scientific value. In the remainder of this paper, I intend to demonstrate how phenomenology is a special kind of EFPA, what is unique to it, and how it may prove valuable in understanding psychopathological phenomena.

## Phenomenological Psychopathology and Explanation: Genetic-Phenomenological Aspects of Experience

Given the task of carving out the essential structures of the different possible conscious acts and their correlative intentional objects, phenomenology has been considered a descriptive discipline. However, in his later works, Edmund Husserl, the founder of phenomenology, presented an additional kind of analysis to complement his descriptive or static phenomenology: genetic phenomenology. Genetic phenomenology is supposed to shed light on the lawfulness by which different temporal moments of experience follow each other and by which more complex conscious objects and modes are constituted over time based on simpler experiences (Husserl, 1966, 1999, 2001). Husserl referred to this genetic analysis, which has been considered to also have a constructive and not purely descriptive character (Kriegel and Williford, 2006, p. 370; Bower, 2014), as “explanatory” phenomenology (Husserl, 1999, p. 318). According to the genetic approach, different temporal moments are not just a series of unrelated events. Rather, any actual moment of consciousness is motivated by and based on prior moments of consciousness. To reveal their lawful connection is the task of genetic phenomenology. Genetic phenomenology, therefore, plays a significant role in the investigation of the constitution of an object or a given type of experience. This form of analysis is unique to phenomenology. Although NIM also highlight the goal of making tacit parts of phenomenal experience explicit, they focus on a given individual’s experience as it is lived through. They might then try to reveal the subjective meaning by which different subjective experiences at time *t_x_* and *t_y_* hinge together. But they do not present a description of genetic laws which govern the temporal unfolding of experience as such and the way different objects and types of experiences arise out of each other.

### Jaspers’ Skepticism Concerning Genetic Understanding and the Explanatory Role of Natural Sciences

Karl Jaspers, protagonist in founding psychiatry as systematic science and pioneer in phenomenological psychopathology, was aware of the distinction between static and genetic analysis. In his famous *General Psychopathology* (Jaspers, 1913, 1963) he emphatically supported the idea that phenomenology as static description of the modes of subjective experiencing has a major role to play in psychopathology. By contrast, he was skeptical about the scientific function of genetic understanding, which he came to deny in later versions of his influential work. In consequence, following Jaspers, most authors in phenomenological psychopathology have focused on static descriptions of abnormal experiences and tried to remain rather cautious about any claims of genetic kind.

However, in the past decade, in numerous publications on that matter, Sass and his colleagues (forthcoming; Sass and Parnas, 2007; Parnas and Sass, 2008; Sass, 2010, 2014) have advocated that alongside static phenomenology genetic phenomenology should be considered when trying to understand and explain psychopathological phenomena. The analyses he offers in the context of schizophrenia provide a more general conceptualization of phenomenology’s possible contributions.

Before presenting his approach, I will briefly sketch Jasper’s highly influential reservations concerning genetic understanding and their underlying theoretical assumptions.

Following Dilthey, Jaspers distinguishes between ‘understanding’ and ‘explanation.’ Unlike Dilthey, however, Jaspers argues that this distinction does not correspond to the division between natural sciences and humanities. It would be wrong, he says, to claim that causal explanations are only to be given in the sciences of the physical realm, while in sciences concerning the psychological sphere only understanding is possible (Jaspers, 1973, p. 253). Rather, he highlights, both understanding and explanation apply to psychology. Explanation in psychology, though, just as in the natural sciences, means to provide insights into causal mechanisms that are non-conscious (“außerbewusst”) (Jaspers, 1973, p. 253). Understanding, by contrast, consists in gaining insight into the process of consciousness or first-person experiencing as it is lived through. Static understanding captures psychological states, their structure and qualities, and how they are experienced from a first-person perspective. Genetic understanding, by contrast, embraces the relationship between different psychological states, that is, how one state emerges out of the other. It refers to motivational rules, for example, how being defrauded results in general suspiciousness (Jaspers, 1973, p. 255).

Now the problem Jaspers sees with genetic understanding is twofold. On the one hand, in most cases, says Jaspers, single tokens of experiences follow each other in a way that is not comprehensible, and therefore a causal explanation of natural-scientific fashion is required (Jaspers, 1973, p. 24). That is, the relationship necessitates an explanation by non-conscious mechanisms when the connection between different moments of experiences cannot be reconstructed by their conscious qualities. On the other hand, even when the relationship between different psychological states can sometimes be genetically understood, the evidence in these cases is merely subjective, requires repeatedly “personal intuition,” presents only “probable” but not “proven” results (Jaspers, 1973, p. 260), and does not itself do any explanatory work (Jaspers, 1973, 253f.). The task of genetic understanding as understanding is restricted to expanding the limits of what is known about consciousness. It brings to light what one has been living through in unthematized manner (“unbemerkt”) but it cannot reach out to what is external to consciousness (“außerbewusst”) (Jaspers, 1973, p. 254). Thus, genetic understanding cannot seize non-conscious causal processes. Rather, by approaching the edge of the understandable it indicates the force of non-conscious causal mechanisms which need to be explained by other scientific methods and theories.

There are two major aspects of Jaspers’ methodological concept that necessitate further consideration. The first is the sharp opposition between understanding and explanation. This includes the assumption that an explanation can only be given by providing insights into natural causality. The second aspect concerns Jaspers’ interpretation of static and genetic understanding. His portrayal of static understanding is highly influenced and oriented by Husserlian phenomenology. By contrast, his delineation of genetic understanding addresses mostly genetic narratives concerning ‘meaningful connections’ between different experiences in a psychoanalytic and therapeutic fashion rather than in keeping with Husserlian genetic phenomenology. His denial of the scientific or explanatory value of genetic understanding is, thus, bound to a certain but also more generic interpretation of genetic understanding.

Both aspects correspond to a couple of important questions that are relevant to demonstrating the explanatory role of genetic phenomenology in psychopathology: Are understanding and explaining two entirely separate methodological steps? Does explanation always and only consist in revealing causal mechanisms responsible for the phenomenon in question? And is there only one form of causality, namely non-conscious natural causality? Is there a different, i.e., a more Husserl-oriented, form of genetic understanding possible that could indeed contribute to the explanation of psychopathological phenomena?

### Sass: The Explanatory Role of Phenomenology in Psychiatric Disorder

Sass answers all these questions differently than does Jaspers. Unlike the pioneer of phenomenological psychopathology, Sass advocates the view that there is not only one kind of causality. Among other concepts, he retrieves Aristotle’s four causes and highlights the variety of possible contributions to explanatory psychopathology (Sass, 2014, p. 372): six possible phenomenological relationships of explanatory significance, of which three are synchronic and three diachronic. He explains them in the context of schizophrenia:

#### Synchronic Relationship: Equiprimordial

In this case, different elements or structures of experiencing are mutually implicating or complementary. Sass gives the example of schizophrenic hyper-reflexivity, i.e., a heightened reflective focus on conscious aspects and diminished self-awareness. Both phenomena correlate given that overly focusing on certain aspects that usually remain in the background of one’s experiencing phenomenally implies a reduction of the sense of being the central pole or subject of experience (Sass, 2014, p. 369).

#### Synchronic Relationship: Constitutive

In this case, explanation is done by demonstrating how a malfunction of the constituting process can effectuate a disturbance at the constitutive level. According to phenomenology, subjectivity is constitutive for or the condition of possibility for world-objects and world-experience as a whole. Disturbances concerning the latter can therefore correspondingly be constituted by disturbances in constituting subjectivity. Again, Sass gives the example of schizophrenia and highlights that “hyperreflexivity and diminished self-presence” entail “a certain disorganization and fading in the field of awareness” (Sass, 2014, p. 369).

#### Synchronic Relationship: Expressive

In this case, explanation refers to revealing the relationship between certain contents of experience and the underlying structure of experience. Sass refers to Tausk’s (1933) classic description of a patient’s delusion of being determined with respect to both experience and action by an “influencing machine” situated in a different room. While this delusional claim seems incomprehensible at first sight, it becomes more understandable when one takes the delusional content to be a manifestation of the underlying experiential structure. Given the diminished self-awareness described in schizophrenia, one’s own experiences and actions can gain an alien character such that they ultimately culminate in a delusion of being controlled by an influencing machine (Sass and Parnas, 2007, p. 80).

#### Diachronic Relationship: Primary/Basic

With this relationship Sass introduces the notion that a given symptom *s_0_* of a pathological condition *p* can be more basic than other symptoms *s_1,2,3…_* involved in *p*. Thus, the idea is that different symptoms may not simply be the direct effect of a common biological cause *c* but have an experiential order such that *s_0_* is caused by *c*, while *s_1_* is not directly caused by *c* but a result or after-effect of *s_0_*. For instance, a neurocognitive malfunction *c* might provoke an unusual salience of tacit sensations which amount to or equiprimordially imply hyper-reflexivity *s_0_*. This hyper-reflexivity might, in consequence, result in a loss of spontaneity *s_1_*: emotions and actions lose the normal automatic character they usually have and can only be effectuated by will power and an effort to draw one’s attention away from the disturbing sensations toward other elements of experience (Sass and Parnas, 2007, pp. 82–83).

#### Diachronic Relationship: Consequential

This relationship is an instantiation of the “Basic/Primary”-relationship in which *s_1_* is a consequence of *s_0_*. In some cases, hyper-reflexivity might not immediately and equiprimordially arise with unusually salient sensations. However, even slightly disturbing sensations might induce an impulse to reflect and scrutinize occurring bits of experiencing and thereby, over time, potentially create the habit to hyper-reflect (Sass and Parnas, 2007, pp. 83–84). Hyper-reflexivity can, thus, be primary (*s_0_*) or secondary, i.e., consequential, to other experiential structures and symptoms (*s_1_*). In both cases, hyper-reflexivity can trigger a cascade of further consequential symptoms.

#### Diachronic Relationship: Compensatory

This relationship is a different kind of instantiation of the “Basic/Primary”-relationship. In this case, *s_1_* is not directly effectuated by *s_0_*. Rather, *s_1_* follows *s_0_* as a compensatory reaction or process. In this sense, hyper-reflexivity can also occur as a compensation for a diminished sense of self (Sass and Parnas, 2007, pp. 84–85). Given that a person does not have a strong self-feeling (*s_0_*), she might activate an overcompensating self-reflective attitude (*s_1_*) to get a better grasp of herself and reduce sensations of self-alienation. Here again, hyper-reflexivity (*s_1_*) can be the basis for further symptoms (*s_1__+__n_*).

His classifications, thus, reflect the view that there exists an experiential or phenomenal causality that refers to the relationship between different elements of experience both at the same point of time *t_0_* (synchronic or static) or at different points of time *t_0_* and *t_0__+__n_* (diachronic or genetic). Accordingly, Sass does not reduce all causality to natural causality. Moreover, unlike Jaspers, he takes experiential causality not simply to be a reconstruction or narrative of how the meaning or content of an experience arises out of past experiences. Rather, the relationships he has in mind are structural and (quasi-)lawful, laden with a different kind and degree of evidence. That hyper-reflexivity and diminished self-affection can be regarded as two sides of the same coin is not just a plausible story but can be given in actual phenomenological intuition by comparing their structures and structural complementarity. The same applies to the example of the influencing machine delusion. Here, the incomprehensible semantic content or meaning of the experience will be compared with potential explanatory experiential structures, as is the case with diminished self-awareness. Describing the experiential structures manifest in a delusional claim, thus, amounts to both understanding and explaining the experience in question.

Another major aspect of Sass’ approach is that phenomenology contributes to the explanation of psychopathological phenomena even when a biological cause is at play. That is, even when, for instance, a symptom like hyper-reflexivity *s_1_* is the after-effect of a neural-based *c* experienced disturbance of sensations *s_0_*, there can be symptoms *s_1__+__n_* following hyper-reflexivity through experiential processes rather than biological mechanisms. And these experiential processes require a phenomenological or experiential explanation. By showing how phenomenology can contribute to explaining such experiential processes, and given his acknowledgment of different kinds of causes, Sass’ genetic approach, which is much closer to Husserlian genetic phenomenology than Jaspers’, can, then, be beneficial for an integrative, multidisciplinary psychopathology.

## Phenomenology and the Cognitive Approach: the Explanatory Role of Phenomenology in Psychological Disturbances

The notion that all psychopathological and psychological phenomena ultimately hark back to natural, i.e., biological causes, which dominated at the time in which Jaspers brought forward his single-cause-theory, still has not completely left the field. However, as early as in the 1970s, the growing criticism of the overly reductionist biomedical model led to a more integrative model: the biopsychosocial model (Engel, 1977). The idea was to introduce psychological, behavioral, and social causes to the model without denying the role of biological causes. Since then, the biopsychosocial model has grown in importance and has become the standard paradigm for today’s mainstream psychopathology. The collaboration of different approaches concerning potential causes of a certain disease was additionally enabled by the changes implemented in the DSM-III (1980), which consisted in the separation of etiology and diagnosis. Since then, and in contrast to prior versions, diagnostic criteria in the famous classification system do not hinge upon certain assumptions concerning the causes that have led to the symptoms in question. Accordingly, an agreement regarding each classification is made. The agreement concerns only what the explanandum is, that is, a certain cluster of symptoms that constitute a psychological disorder such as major depression (MD).

Once such agreement has been found, the different approaches can contribute to a multidisciplinary, multi-causal, multi-methodological, and ultimately integrative theory of psychopathological phenomena, the explananda.

In fact, in the biopsychosocial model, EFPA alongside other approaches find their place just as Sass’ account suggests. However, in the past decades, despite its explanatory potential, phenomenology has mostly contributed to the task of describing and defining what the explananda of psychopathological phenomena are. Predominating in giving experiential or psychological explanations of psychopathological phenomena has until now been another EFPA: CT. Given its focus on observational data – be they third-personal in kind (behavior) or first-personal reports (experience and cognitions) – and general orientation in natural-scientific methodology, CT has been widely acknowledged and become the major representative of EFPA in the biopsychosocial paradigm and mainstream clinical psychology.

Despite the merits of CT in establishing EFPA against traditional reductionist biomedical accounts, there are significant limitations to CT. In what follows, I will sketch some of these limitations and argue that phenomenology, genetic phenomenology especially, complements CT.

### The Cognitive Approach as an EFPA

Like the biopsychosocial model, the cognitive approach to psychopathology has a pragmatic view on the etiology of psychopathological conditions. It consists in the notion that there are manifold processes and factors “responsible for the development, maintenance, correction, and prevention of psychopathology” (Alford and Beck, 1997, p. 42). The cognitive approach concerning these manifold processes and factors involves the following main features:

#### Open Causal System

Cognitive Theory assumes that different forms of causes exist that can lead to a psychopathological condition such as MD. It fully accepts that to be able to give an exhaustive account of what causes depression, one would have to consider “for example, hereditary predisposition, faulty learning, brain damage, biochemical abnormalities, etc., or any combination of these” (Beck et al., 1979, p. 19). However, this does not imply that ultimately only biological causes are taken to explain psychopathological phenomena, for accounts that reduce explanation to revealing “efficient causation” are “reductionistic or atomistic in their metaphysical assumptions” (Alford and Beck, 1997, p. 39). Moreover, given its view that the subject is a “free agent” (Alford and Beck, 1997, p. 40), CT holds that psychopathological phenomena “are at a level of complexity or interrelatedness that generally does not lend itself to efficient causal analyses” (Alford and Beck, 1997, p. 39). It is also not the goal of the cognitive model to “address itself to the question of the possible ultimate *etiology* or cause” (Beck et al., 1979, p. 19) which “would require the inclusion of the ‘Big Bang’ plus all prior and subsequent events” (Alford and Beck, 1997, p. 40). However, the fact that CT can only give a partial causal explanation does not diminish its value, for “scientific analyses of complex open systems always remain incomplete” (Alford and Beck, 1997, p. 40).

#### Explanatory First-Person Approach (EFPA)

Unlike biological and behaviorist accounts prevailing in the first half of the century, the cognitive approach, increasingly popular in the 1960s and 1970s, explicitly acknowledged the causal power of conscious experience: “[A] person’s conscious phenomenal experience (perception) can take on an emergent causal status. Thus, it is equally reasonable to ask, ‘What causes consciousness?’ and ‘What does consciousness cause?”’ (Alford and Beck, 1997, p. 42). Accordingly, after many decades of predominant study of the unconscious (psychoanalysis), biological processes (medicine), and behavior (behaviorism), the analysis of conscious experience had become an essential part of explaining psychopathological phenomena, as the following quotes indicate:

“*Internal (phenomenological) and external (environmental) dimensions are integrated into the fundamental philosophical position and theoretical constructs of cognitive therapy*.” (Alford and Beck, 1997, p. 107)
“*The phenomenological approach is a core component of cognitive theory* […].” (Alford and Beck, 1997, p. 129)
“*Cognitive theorists simply seek to obtain a more complete picture of this representation (learning) through attention to the content of idiosyncratic, phenomenological perceptions of relationships among events*.” (Alford and Beck, 1997, p. 129)

For CT, the way a person structures the world and the events happening in it are causative regarding the person’s affects and behavior (Beck et al., 1979, p. 3). And the idiosyncratic ways of structuring the world are themselves said to be phenomenal aspects of the person’s lived experiences. For, according to the cognitive approach, the organization of how an individual takes the world and its events to be is not only grounded on “reality” itself (Alford and Beck, 1997, pp. 22–24) but essentially determined through the *meaning* by which an individual apprehends world events and the relationship among them in the first place. And, opposing the psychoanalytic claim that these world-structuring meanings are not directly accessible, CT highlights that “the meanings were found to be available through introspection, and not to require the penetration or circumvention of a wall of repression in order to be elucidated” (Alford and Beck, 1997, p. 109; Varga, 2014, p. 176).

In all, CT is, thus, entirely an experiential account: The structures *s* which render an experience *e* as having the phenomenal quality *p* or *q* are themselves phenomenal aspects of *e* or can be given in experience by reflecting on one’s experiences. The task of CT, then, is to reveal *s* and the determining, constitutive or causative relationship *r* they entertain with *e* as well as to demonstrate how a certain instantiation of *s* correlates with *p* or *q.*

#### Cognitive Primacy

CT further specifies this general formulation of the main idea of the accounts which I have referred to as the group of EFPA. In fact, CT holds that the structures *s* which organize the lived experience of an individual are *cognitions*, which consist of beliefs and automatic thoughts such as internal verbalizations and mental images (Taylor, 2006, pp. 16–17). It is by means of cognitions that meaning is possible in the first place. “These cognitions constitute the person’s ‘stream of consciousness’ or phenomenal field” (Beck et al., 1979, p. 8). The “meaning-making structures of cognition” (Alford and Beck, 1997, p. 15) are termed *schemas* which “provide the ‘structure’ for both phenomenological experience and interrelated cognition, affect, and behavior” (Beck and Alford, 2009, p. 257). This means schemas “constitute the explanatory terms for the organization of psychological activity and of phenomenological experiences” and “influence the emotional, behavioral, and physiological aspects of the various psychological disorders” (Beck and Alford, 2009, p. 257). Given that cognitive schemas organize experience, CT takes dysfunctional cognitions to underlie abnormal experiences. It is due to faulty cognitive organization that experiences gain a pathological character which is why, through the lens of CT, mental disorders are to be conceived of as “thinking disorder(s)” (Beck and Alford, 2009, p. 208).

The disturbance in thinking can refer to either (a) “cognitive content” or (b) “cognitive processing” (Beck et al., 1979, p. 16) or both:

(a) The former, relating to “meaning,” concerns one’s constructions of one’s own “self, the environmental context (experience), and the future (goals), which together are termed the *cognitive triad*” (Alford and Beck, 1997, p. 16). All psychopathologies come with a certain set of beliefs concerning these three aspects. For instance, in the case of depression, assumptions about the self, environment and future are characterized by negative beliefs.

(b) Cognitive processing, relating to “meaning elaboration” (Alford and Beck, 1997, p. 16), concerns the way situational information is being processed and integrated into the person’s cognitive structure. Faulty interpretations of available information, according to CT, might then give rise to problematic and erroneous beliefs prone to entail pathological affects. Thus, it is not always the information as such that induces pessimistic beliefs in the sense of (a) but rather how information is cognitively organized. For instance, in depression, “neutral or even favorable events are processed in such a way as to produce a negative conclusion” (Beck and Alford, 2009, p. 233). Examples for maladaptive and inadequate information processing are selection biases and overgeneralization. A selective focus on a single negative aspect of an otherwise positive event might lead to an inadequate pessimistic assessment of the whole event. Furthermore, a single negative event – adequately adjudged as such or not – might lead to beliefs concerning reality more generally: An unfortunate encounter with one person might result in the erroneous and overgeneralized conclusion “No one likes me” (Beck et al., 1979, p. 14; cf. Pretzer and Beck, 2014, p. 55).

Thus, according to CT, the relationship between cognition on the one hand and emotion, mood, and affect on the other hand is characterized by the primacy of cognition over the latter. This causal primacy of cognitive content and processing can be understood in either *temporal* or *constitutive* terms. Sometimes thinking processes temporally and discretely antecede moods, emotions, and affect that directly result from the thinking processes. In other cases, cognitions form constitutive parts of a token experience *e* with a certain emotional or affective quality *q*, whereby cognitions render *e* as having the phenomenal quality *q* in the first place.

It is noteworthy that there exist alternative cognitive approaches that, influenced by evolutionary epistemology and attachment theory, have highlighted the affective dimension of thinking and belief (Balbi, 2008, pp. 21–23). According to the Cognitive Post-Rationalist model (Guidano, 1991), feelings and emotions present the evolutionary antecedent systems and structurally primary carrier of beliefs. Accordingly, in contrast to standard CT, this model proposes a primacy of affectivity. Here, however, I will focus on standard CT given that it is far more widespread and considered mainstream.

#### Integrative Power and Commitment to Quantitative Empirical Methods of Psychology

Despite the axiomatic character of cognitive primacy and the emotion theory involved, proponents highlight that CT’s basic assumptions are not static and unchangeable. Rather, the principles of CT themselves are constantly to be challenged by future “tests” as the major proponents of CT, with Popper in mind, emphasize (Alford and Beck, 1997, p. 17). CT, thus, considers itself an open system that theoretically and conceptually adapts as needed. The necessity for modifications of this kind, however, can only be adjudicated relying on tests of a special kind, namely those developed by quantitative psychology. According to CT, tests that qualify buttressing or falsifying conceptual models of cognitive theory, are, then, based on quantitative-statistical methods. By using operational definitions, conceptual assumptions are translated into hypotheses to be challenged by empirical observations. CT considers this one of the main criteria for guaranteeing the scientific status it attributes to the cognitive approach and its concepts.

By the same token CT ascribes an integrative role to itself concerning the organization of different psychotherapeutic and psychopathological approaches in one system of psychotherapy. The general integrative rationale and agenda of CT can be formulated as follows: (a) Conceptual models of CT have been successfully tested for empirical validity; (b) future modifications of cognitive concepts will only be motivated by potential new empirical findings; (c) other approaches can complement CT if they are testable by quantitative-statistic methods and their empirical validity has been shown; and (d) other approaches for which empirical validity has been shown shall be integrated into CT by translating their concepts into cognitive terms (cf. Alford and Beck, 1997, pp. 109–112). To conclude, CT is an “integrative paradigm” (Alford and Beck, 1997, p. 112) of psychotherapy itself, committing itself and the discipline of psychotherapeutic science to the quantitative-empirical methods of psychology.

### Limitations of the Cognitive Approach in Explaining Psychopathological Phenomena

In the last decades, CT has admittedly become a major pillar in the building of psychotherapy and is considered mainstream in clinical psychology. Even though other approaches to psychotherapy have not ceased to exist, CT has become a widely used framework applied to describe the psychological processes involved in psychological disorders. Through the lens of consciousness research, CT has earned many merits by establishing the idea that conscious processes do play a role in the explanatory science of psychological and psychopathological phenomena. These merits derive directly from the main characteristics of CT listed in the previous section, for it is the assumption of a plurality of causal factors that secures consciousness and its processes a place in the project of dismantling etiological pathways to certain psychological conditions. Moreover, CT highlighted the explanatory relevance of conscious processes for psychopathological phenomena by demonstrating these effects through quantitative tests based on empirical methods. Given the acceptance of quantitative empirical methods in the broader scientific community, CT succeeded in increasing the acknowledgment of conscious processes as scientifically and especially causally relevant – even among proponents of reductionist approaches which place emphasis on the (neuro-)biology of psychopathology. By the same token, CT introduced and consolidated quantitative empirical methods in psychotherapy and clinical psychology. Given the affinity between these methods and cognitive concepts, CT as a theoretical framework could establish and further amplify the central role it plays in psychotherapy and clinical psychology. Three different aspects are, then, deeply interconnected with the success of CT: (1) the broader acceptance of the explanatory role of conscious processes in psychopathology; (2) the introduction of quantitative empirical methods as the standard of psychotherapy research and clinical psychology; and (3) the increasing dominance of cognitive concepts and CT as a main theoretical framework in psychotherapy research and clinical psychology.

Despite its undoubted merits for consciousness research, it is an open question whether this threefold alliance between the study of consciousness, quantitative empirical methodology, and cognitivism exhausts all that can be said about conscious processes involved in psychopathological phenomena. In the following, I will present some limitations of CT. In doing so, my aim is to formulate a critique not of the cognitive approach *per se* but of the rather universal claim that CT is the main integrative paradigm and theoretical framework for the study of conscious processes in psychopathology. I will further argue that it is phenomenology, particularly genetic phenomenology as described in the previous sections, that can significantly complement cognitive theory and its conceptual and methodological approach to assure a better understanding of the experiential processes involved in psychopathological phenomena. To corroborate my argument and without neglecting the possibility that there might still be more limitations to the cognitive approach (see e.g., Varga, 2014), the following list is restricted to problematic aspects of CT that are salient from a phenomenological perspective and could also benefit from a complementary phenomenological view.

#### The Development of Schemas

CT describes schemas as being responsible for the cognitive structure on the basis of which information is processed. In this way, given a certain situation *s* in an environmental context *ec*, i.e., *s_ec_*, schemas determine a person’s cognitions, emotional resonance, and behavioral reaction based on *s_ec_*. Put differently, schemas render a token experience *e* related to *s_ec_*, i.e., *e (s_ec_)*, as having the phenomenal quality it has. *E* (*s_ec_*), moreover, leads to certain actions because of the phenomenal quality it has. In the last decades, CT has, quite successfully, spent tremendous effort in investigating the different kinds of schemas underlying different psychopathological conditions and how they might be altered.

Surprisingly, however, given the major importance ascribed to schemas, only little attention has been paid to the question of how these schemas arise over time, which factors underlie the formation of schemas, and how these factors interact so that they lead to “relatively permanent or habitual ways in which an individual interprets and reacts to the environment” (Beck and Alford, 2009, p. 257). The position of CT concerning the formation of schemas is rather generic and vague:

“*The precise manner in which the negative cognitive bias has evolved over time, and the circumstances that selected this particular cognitive programming, may never be entirely explained, although evolutionary processes presumably selected such mechanisms in the same manner as other adaptive mechanisms are selected*.” (Alford and Beck, 1997, p. 40)

In a more recent depiction of “the generic cognitive model” (Beck and Haigh, 2014), CT places emphasis on genetic parameters that cause physiological activity that entails attentional biases. These attentional biases ground a faulty representation of the situation in which they occur. Given the wrong representation of the situation, then, attentional biases are followed by memory biases, for when situations are interpreted in a faulty way they become stored in a distorted manner. Over the course of time, erroneous storing of past events consequently becomes habitualized and petrified in inadequate beliefs, which constitute maladaptive cognitive schemas. Maladaptive cognitive schemas motivate interpretative biases, which, in turn, cause faulty information processing and attentional biases in future situations.

CT, however, does not always stress biological factors in the formation of maladaptive schemas and schemas in general (Pretzer and Beck, 2014). Rather, CT highlights that schemas are a consequence of previous experiences. The encounter of an individual with an object or situation *os_0_* motivates the formation of a concept *OS* which determines how future similar objects or situations *os_1,2,3,…_* are apprehended.

All in all, despite ascribing natural subpersonal factors a potential role in the formation of schemas, the main mechanisms through which schemas are built involve phenomenal experience: Attentional biases and other primary experiences with objects and situations lead to distorted memories of past events, which by habitualization processes are transformed into schemas that determine how an individual perceives her environment. Attentional biases due to biological causes such as genetic predisposition for inadequate physiological activity, then, present only a specific case of a wider range of possibilities of causes for the distorted perception of a certain situation. Obviously, schemas themselves are said to play a major role in the distortion of perception. Indeed, new schemas, call them *secondary* schemas, can arise on the ground of interpretative biases due to existing schemas, call them *primary* schemas. However, the important question is which other possible reasons might induce distorted perceptions of a set of situations which then entail the formation of maladaptive schemas, whether CT offers a system of potential reasons for distorted perceptions and whether such CT-based system is exhaustive. For my purposes here, it suffices to answer only the second and third part of this question and my answer is negative in both cases. First, CT does not provide a system of reasons, factors or processes involved in the *formation* of schemas but focusses on how schemas underlie distorted perception and how they might be altered. Second, I doubt that CT has even the means to decide whether its system is altogether exhaustive. At this point, I have only demonstrated that there is a *factual* limitation to the cognitive approach in clarifying how schemas develop. In Section “CT, Introspective Data, and Quantitative Empirical Psychology,” I will make a stronger claim and argue that, given its methodological repertoire, CT is inapt to grasp all possible processes underlying distorted perceptions, that, furthermore, it does not have the means to decide whether its list is exhaustive, and that therefore its limitation in clarifying how schemas develop is *principle*.

#### The Nature of Schemas

A somewhat limited view is also salient concerning the nature of schemas. CT provides a clear definition of what schemas are in terms of their function: schemas structure an individual’s conscious thought, affect, and behavior, i.e., her experience. Defining *x* by referring to its function, though, is not the same as explaining what *x* consists of. According to CT, schemas consist of beliefs, which may be beliefs about oneself, others, situations, behavioral strategies, and other kinds of object-related beliefs. In fact, this is one of CT’s main assumptions and, CT has repeatedly stressed that its assumptions and concepts have been proven empirically (Alford and Beck, 1997, p. 100).

One objection, however, is that the belief-assumption, i.e., that schemas consist of beliefs and of beliefs alone, is fundamental and philosophical and that the belief-assumption, as such, cannot be proven empirically (Varga, 2014, p. 167). It is one thing to empirically show that beliefs are significantly involved in structuring a person’s phenomenal experience. It is another thing to demonstrate that schemas consist in *nothing but beliefs*. Put differently, CT might be right in claiming that beliefs shape our experience in most cases. It does not follow from that, though, that there could not be non-judgmental or pre-predicative processes which contribute to a schema’s function of structuring a person’s experience. The question, therefore, arises as to whether CT, given its methodological repertoire, has the means to decide whether beliefs exhaust the nature of schemas. I will later argue that, for methodological reasons, CT *cannot* give a final answer in principle (see CT, Introspective Data, and Quantitative Empirical Psychology). Here, I will focus on the possibility of non-judgmental or pre-predicative processes involved in the structuring of an individual’s phenomenal experience.

In recent phenomenological literature, it has in fact been argued that non-propositional horizons might be presupposed by beliefs with propositional content, rendering beliefs possible in the first place (Ratcliffe, 2015, pp. 143–154, esp. 146). Ratcliffe (2008) terms these pre-predicative horizons “existential feelings.” His approach draws from a general distinction well-established among phenomenological thinkers such as Scheler, Heidegger, Sartre, and Ricoeur: mood and affect. While affects are intentionally directed at certain objects or facts to which they are responses, moods do not refer to a discrete object (Rosfort and Stanghellini, 2009, p. 258). Rather, they determine the field of awareness in which intentional objects may appear and how they do so. In this vein, Ratcliffe describes existential feelings as the background against which the experience of certain possibilities arises. Put differently, given a certain existential feeling *ef_1_*, an individual’s phenomenal experience is characterized by the different kinds of possibilities related to *ef_1_*: for instance, what kinds of events seem possible; what kinds of cognitive, affective, and behavioral responses to possible events are possible; and what kinds of cognitive, affective, and behavioral responses of others to one’s own possible actions and possible events are possible. And these possibilities differ from those intrinsic to another existential feeling *ef_x_*. Given the different possibilities inherent in different existential feelings, phenomenal experience is different in each case, i.e., existential feelings structure an individual’s phenomenal experience.

One example would be the existential feeling of guilt in depression, “existential guilt” (Ratcliffe, 2008, pp. 128–154), which is distinct from the feeling of guilt related to a specific deed or event. Existential guilt consists of a basic feeling of being “guilty as such” (Fuchs, 2003, p. 238). Based on this background, single “experiences of *intentional* guilt” (Ratcliffe, 2008, p. 145) referring to specific events might be tokened. The crucial point is that existential guilt is “something that does not rest on any kind of judgment (moral or otherwise) regarding one’s deeds” (Ratcliffe, 2008, p. 145). Rather, given that existential feelings are taken to be enabling beliefs as such (Ratcliffe, 2008, p. 146), existential guilt determines what kinds of beliefs are built and how, for existential feelings also constitute the way in which feelings are interpreted and expressed. Existential guilt, then, makes one’s narratives of world events and the specific feelings involved include guilt-related beliefs. Thus, according to this view, it is a basic feeling of being guilty that is constitutive of a depressive person’s proneness to form guilt-related beliefs. And not the other way around.

This is not to say that beliefs play no role in structuring a person’s phenomenal experience nor that changing one’s beliefs does not affect “how a person finds herself in the world” (Ratcliffe, 2008, p. 146). Rather, this example simply demonstrates that there might be pre-conceptual schematizing processes which structure an individual’s phenomenal experience without deriving their “force” (Beck and Alford, 2009, p. 257) from an underlying belief. Furthermore, it could be that beliefs related to a certain schema arise on the basis of existential feelings in the first place, i.e., that schemas are in fact existential feelings and that beliefs are secondary to them.

To conclude, from a phenomenological perspective, one might hold that CT got it right in highlighting an essential relationship between schemas, the structure of phenomenal experience, beliefs, and feelings, but that its depiction of this relationship is non-exhaustive.

#### Cognitive Primacy

Obviously, the latter and all of what has previously been said so far regarding the limitations of the cognitive approach bears significantly on CT’s assumption of cognitive primacy. If it has not yet been sufficiently clarified how certain schemas evolve, then maybe some or all beliefs, which the schemas consist of, depend on non-cognitive structures. This does not preclude beliefs generally determining a person’s further phenomenal experience and having a functional role as schema. But it could be wrong to ascribe primacy to beliefs over feelings altogether. It could be that a certain feeling *f_1_* induces reflective and interpretative processes, which eventually lead to forming a belief *b*, which in turn induces the feeling *f_2_.* This means *b* is secondary to *f_1_*, but primary to *f_2_.* It could also be that *f_1_* would never lead to *f_2_* if it were not for *b* as a mediator. Given the numerous studies conducted by CT, one can indeed say that beliefs are causative with regard to feelings and phenomenal experience in general. But since the development of schemas and beliefs is not sufficiently clarified, all that these studies show is the *relative causative cognitive primacy* of the specific schemas and related beliefs under scrutiny. They do not demonstrate that all feelings result from underlying beliefs.

A similar conclusion can be drawn regarding the nature of schemas. CT has gathered proof in numerous studies that beliefs can have the function of schematizing an individual’s phenomenal experience. However, CT has not revealed the nature of schemas exhaustively. It has not shown that all that schemas consist of are beliefs. Put differently, CT’s findings do not suffice to clarify whether it is only by means of beliefs that schemas fulfill their function in structuring phenomenal experience. All that CT’s studies prove is *limited constitutive cognitive primacy*: Some schemas might primarily structure phenomenal experience through certain beliefs, and/or beliefs play the major part in the schematic structuring of phenomenal experience. Limited constitutive cognitive primacy, thus, is compatible with both (a) schematizing structures or even full-blown schemas exist that do not contain beliefs at all and are pre-cognitive and (b) there might be schemas that contain both beliefs that do most of the structuring work *and* non-cognitive elements that contribute to the structuring function of schemas.

The question, then, arises as to whether CT could run further studies to corroborate its stronger claims. In the next section, I will argue that CT has not the means to vindicate *absolute causative cognitive primacy* and *exhaustive constitutive cognitive primacy* altogether.

#### CT, Introspective Data, and Quantitative Empirical Psychology

As I have indicated before, the reason why CT cannot provide a final answer regarding cognitive primacy lie in CT’s methodology. The problem is not so much with the quantitative empirical approach *per se* but with the assumptions CT makes with regard to introspective or phenomenological data to be obtained. The assumptions concern introspection as both a feature of consciousness and scientific access to consciousness (cf. Feest, 2012).

First, CT “maintains that there is a representation of propositional content involved” (Varga, 2014, p. 170) in our thinking such that every occurrent thought carries a mental representation of a propositional content *p*. Based on this, CT further assumes that by having a person report her thoughts one can gain insight into the person’s lived experience. However, as Varga (2014, pp. 169–174) argues, the utterance ‘I think/thought *p*’ can have different meanings which need not be merely representational. The utterance does not necessarily entail ‘the thought *p* occurs/occurred to me.’ In some cases, ‘I think/thought *p*’ is simply *explanatory* of one’s actions but not a representation of one’s lived experience. Referring to an example of Varga’s (2014, p. 171): The cabdriver who justifies his stopping at a red light might say ‘I thought the light was red.’ But this does not necessarily mean that, when stopping, the cabdriver went through a thinking experience ‘the light is red.’ Rather, he might have had a perception of the light as being red and stopped. Moreover, the utterance ‘I think *p*’ could be *expressive* of a set of feelings and emotions: ‘I thought I would die’ could simply be an expression of an episode of strong fear and all different feelings involved.

Taking self-reports, which often have a propositional form, to be directly representative of a person’s lived experience distorts the conceptualization of the latter. It places too much emphasis on the role of propositional thought and belief, when in fact uttering ‘I think/thought *p*’ does not in all cases equate to having the conscious thought *p* passing through one’s mind.

A second assumption refers to the relationship between conscious thoughts and inner speech. CT holds that cognitions are accessible through automatic thoughts, which are manifest in inner speech and/or conceptually structured mental imagery (Sheldon, 1995, pp. 150–151; Safren et al., 2000, p. 328; Taylor, 2006, pp. 16–18; Gilson et al., 2009; cf. Varga, 2014, pp. 173–176), and therefore accessible to individuals undergoing them (Riskind, 2006, p. 63). While it might be true that many thoughts are accompanied by resounding words in foro interno, the crucial question is what implications this has for the relationship between inner speech, conscious thinking/thought, feeling and lived experience as a whole. Hence, the question is whether the phenomenon of inner speech corroborates the thesis of cognitive primacy.

While in some cases the resounding of words in inner speech might constitute one’s current thinking process, in many others it merely accompanies a thought or a result of prior experiencing processes. For instance, having a fight with members of your family, you may undergo feelings of bodily distress, anger, fear of loss, sadness, hopelessness and the like. Eventually you will come to hear the words ‘it’s all your fault’ resounding in your mind. In many cases, rather than presenting the thinking process itself, these words are an attempt to regulate one’s emotions and make sense of them ex post: The belief ‘it’s all my fault’ might allow one to explain fears of loss and sadness; further it might allow one to forget one’s anger about the other’s behavior and to regain a positive view of the beloved person, because if it was all my fault the other is ultimately excused for her unacceptable behavior; it might, in consequence, allow for one to regain a sense of hope because if ‘it’s all my fault’ then I can behave differently in the future to prevent disputes of such kind and feel in control of my future with the other. Hence, the resounding of ‘it’s all my fault’ does not grasp the full content of one’s lived experience while present in one’s mind. Moreover, it could be that, rather than coming to one’s mind via an inner utterance, the belief ‘it’s all my fault’ is manifest as a feeling of deep guilt overshadowing the prior feelings mentioned above. This feeling of guilt could be accompanied by the inner utterance ‘it’s all my fault’ *or not*. If it is, then the reason for this could be either that the inner utterance immediately triggers guilt-feelings or that the accompanying utterance expresses guilt-feelings. However, there is even the possibility that these words run through my head without being related to guilt-feelings. They may just present the thought that it is possible that it is all my fault without evoking any further consequence: I internally hear the words, but I do not *feel* them to be right and let them pass. In this case, the words partially constitute my thinking while they occur, but they are far from being the whole story. My lived experience rather embraces an emotional detachment from the words which run through my head. My not feeling committed constitutes my lived experience just as do the words.

Now, already this quite simple example demonstrates that the phenomenal fact of internal resounding words alone does not allow a clear statement on the relationship between thought/thinking and feeling – and not even between conscious thought/thinking and inner speech.

How else could CT gain access to a person’s thoughts, feelings and their relationship? There seems to be a solution. One could accept that automatic thoughts are not always manifest as internal words and that inferences from a person’s descriptions of her feelings to the person’s thinking process is not possible, but claim that the person herself has direct access to her own lived experience nonetheless. Lived experience could be taken as a more complex phenomenon embracing thinking and emotional processes. One could then try to have a person identify with statements concerning her thoughts and feelings. As to thinking, the instruction could be that the person does not have to simply read off or speak out internal verbalizations of automatic thoughts but should take all happenings of her conscious flow into consideration when being confronted with certain written propositions that reflect one belief or another. In this case, the person should indicate with which belief-statements she can identify or not. Granting possible introspective mistakes concerning incorrect identifications with certain beliefs and to reduce error, one provides varying statements representing each belief. Then, even though thoughts might not necessarily resound in internal speech, the propositional content or beliefs involved in one’s lived experience can be identified. Then one applies a similar approach to feelings.

Furthermore, this being indeed a main rationale for CT’s research, by gathering data from a high number of persons in the same way and subjecting the data to statistical analyses, more general conclusions about the correlation between certain beliefs and feelings can be drawn.

However, this method presents nothing more than part of a solution. It might circumvent the problem that propositional content involved in one’s experience does not always get spelled out internally. Admittedly, it is nonetheless apt to identify beliefs related to certain feelings. But, most importantly, it fails to illuminate the precise relationship between beliefs and feelings. It would be sufficient to work out correlations between beliefs and feelings if the assumption of absolute cognitive primacy were already warranted. Then one could infer from their demonstrated co-existence that a belief is causing or constituting a feeling. But absolute cognitive primacy is precisely in question. Even if CT succeeds in showing that changing an existing belief *b* effectively mitigates an existing feeling *f_1_* or induces a different feeling *f_2_*, nothing follows from that concerning feelings *f_0,-1,-2,…_* which *b* might be an expression or result of. The explanatory power of CT is intrinsically restricted to relative causative and limited constitutive cognitive primacy. This is not a problem *per se* and does not diminish the value of CT-informed psychotherapy (Varga, 2014, p. 184; Ratcliffe, 2015, p. 73). But it becomes an issue when CT interprets its findings as a demonstration of absolute cognitive primacy. Presupposing the latter, CT overestimates the role of beliefs from the start and excludes pre- or non-cognitive factors from its research. However, such factors might be conducive to schematizing an individual’s lived experience – be it directly or by motivating the formation of a certain belief.

### Phenomenology and Non-cognitive Factors: Embodiment, Temporality, Passivity

Although CT has referred to phenomenology as an important resource for CT, it is precisely from a phenomenological perspective that doubts arise about the cognitivist depiction of beliefs, feelings, and their relationship. Put differently, CT’s assumptions about experience conflict with descriptions obtained through phenomenological reflection. Moreover, while CT stresses the significance of *active* thinking processes of the subject, phenomenological psychopathology has a tradition in focusing on *passive* constitution processes which structure an individual’s phenomenal experience and are taken to be the condition for active processes such as ‘judging that *p*’ (Husserl, 1973, 1981, 2001). These passive processes and structures such as embodiment, temporality, intersubjectivity or pre-reflective selfhood are, therefore, pre- or non-cognitive factors that schematize phenomenal experience without necessarily involving concepts. Deviations concerning these interrelated processes and structures have been identified in the context of psychopathological phenomena (e.g., Fuchs, 2001, 2003, 2013a,b, 2014; Parnas et al., 2005; Ratcliffe and Stephan, 2014; Ratcliffe, 2015; Sousa, 2015; Sass et al., 2017; Stanghellini et al., 2016, 2017; Doerr-Zegers et al., 2017).

Despite these differences and apparent conflicts, I suggest interpreting CT and phenomenology as complementary, for relative causative and limited constitutive cognitive primacy are compatible with the view that passive, non-cognitive processes significantly contribute to structuring lived experience. Moreover, active and passive processes do not belong to independent phenomenal realms. Actively applying a concept and ‘thinking that *p*’ involves passive processing of the temporal unfolding of consciousness. Passivity pervades activity; both constitute an individual’s phenomenal experience together. Forming beliefs is based on underlying passive processes (Husserl, 1973). The phenomenological analysis of the latter might, therefore, inform CT by illuminating how certain beliefs arise.

In the remainder of the paper, I offer a brief example in the context of depressive experiencing. Phenomenological psychopathology provides a large body of literature on the plenitude of phenomena involved and highly nuanced insights into the different types of depression (e.g., Fuchs, 2001, 2003, 2013a,b, 2014; Ratcliffe and Stephan, 2014; Ratcliffe, 2015; Doerr-Zegers et al., 2017; Stanghellini et al., 2017). Here, however, I will focus on the development of beliefs. Rather than giving a full phenomenological description of the depressive experiencing in question, my aim is to show that circumstances under which depression-related beliefs emerge may vary. To be more specific, regarding etiology, phenomenology, type and therapy of depressive experiencing, it might matter what kind of experiences and related processes of experiential unfolding negative beliefs are based on. Accordingly, I want to defend the claim that the analysis of passive structures involved in the genesis of beliefs is indispensable. This holds true even if one were to follow the cognitive approach and direct one’s clinical focus – conceptually and therapeutically – on beliefs as the ground of psychopathology.

To substantiate my claim, I will provide an exemplary description of how depressogenic beliefs in the context of burnout may arise. According to mainstream clinical psychology, burnout is not a distinctive nosological category, which is reflected in the fact that it is listed in neither DSM-V nor ICD-10. Rather, it is considered to fall under the category of MD. Moreover, it has been argued that burnout does not differ from MD regarding symptomatology (Bianchi et al., 2013, 2015) and cognitive styles (Bianchi and Schonfeld, 2016). Proponents of burnout, however, conceive of burnout as a process that may ultimately result in MD as the endpoint of a downward spiral (Burisch, 2006, pp. 39–40). So, the question is whether the antecedent burnout-process has a clinically significant phenomenal impact on the way the MD it entails is experienced. Those equating burnout with MD will hold that it does not. This view, I think, can be challenged.

Obviously, the best way to do so would be to provide a fine-grained phenomenological comparison of burnout and non-burnout MD. Since my aim is to show the significance of phenomenological analyses under the premises of CT, I will set the issue of a full-fledged phenomenological investigation aside and focus on beliefs. According to CT, MD is based on a set of beliefs coined the negative cognitive triad (Alford and Beck, 1997, p. 16). ‘I am worthless,’ ‘It is hopeless, nothing will change,’ and ‘I will never be happy’ are such beliefs that structure an individual’s phenomenal experience and render it depressive.

How could such beliefs arise as a result of the burnout-process? To give an example, I draw on Behnke’s (2009) concept of “bodily protentionality” which consists in the notion that our embodied experience is pervaded by an implicit and pre-predicative expectancy of changes in one’s bodily posture, for our bodily being is characterized by a basic feeling of ‘I can’ presenting a fundamental motility and spontaneity presupposed by all subjective experience (Behnke, 2009, p. 192). Behnke argues that this proto-activity involves the passive expectancy of an on-going possibility of motility including expected shifts in one’s bodily practice. This applies even if we try to hold a certain posture. While doing so, a plethora of bodily shifts occurs to allow for maintaining the bodily posture. Accordingly, there is an inherent striving toward constant shifts in our embodied consciousness which also applies to thinking processes. Moreover, this striving toward changes increases if it is constantly disappointed.

Being in a work attitude the embodied consciousness of a person may passively strive toward such changes. These anticipations will be constantly disappointed when the individual keeps working. In the beginning, in periods of overwork disappointments may be overshadowed by professional success and recognition from other persons. On the level of belief, no problems arise. However, there is a critical point at which disappointments in bodily temporality cannot be compensated anymore. Despite high ambition and goal-directed attitude, professional goals lose their enticing character, as might private components of the person’s life. This need not come with immediate changes in belief. The person might remain in the same work attitude and continue having vocational success. Beyond a critical point, however, success and other positive components such as seeing one’s children grow may lose their fulfilling character and felt meaning. This may induce feelings of self-alienation and guilt toward one’s children. Moreover, the ongoing disappointment of the striving toward a non-work attitude may become habitualized in the sense that the disappointment is passively anticipated together with the feelings of striving, triggering feelings of despair and loss of hope as such (Ratcliffe, 2013). On the ground of such experiences, negative beliefs may develop and foster cognitive styles that entail further depressive experiencing.

Does it matter that the negative beliefs linked to the depressive experiencing stem from an on-going disappointment of anticipations in bodily temporality? There are multiple reasons why it might. First, it demonstrates that beliefs are sometimes secondary to depressive feelings of which beliefs can be an expression rather than the feelings’ primary ground. Second, the striving towards fulfillment in the sense of a desired and anticipated shift in embodied attitude may persist after its ongoing disappointment has triggered depressive experiencing. That is, the striving may remain a phenomenal aspect of lived experience. Third, in consequence, burnout-depression and MD may have a different phenomenal structure, despite similar beliefs and related symptoms. Hence, to distinguish both requires careful analysis of the precise phenomenal structure of lived experience in both conditions to be pursued in future research, which ultimately might also entail ramifications for therapy. Particularly genetic phenomenology and the analysis of passive, pre-cognitive structures and processes lend themselves to such an analysis and thereby significantly complement CT, which has severe blind spots concerning the genesis of beliefs and non-cognitive factors.

## Conclusion

The main goals of this paper have been to introduce the notion of EFPA as a category embracing a variety of accounts that take structures and processes of consciousness to be of explanatory value concerning conscious phenomena, to distinguish phenomenology from other EFPA by describing genetic phenomenology, and to illuminate how genetic phenomenology as the unique feature of phenomenology can contribute to the explanation of psychopathological phenomena. The argumentative strategy consisted in showing how two scientific mainstream approaches, biological reductionism in the case of psychiatric disorder and Cognitive Theory in the case of psychological disturbances, in explaining psychopathological phenomena could benefit from complementation by phenomenology.

In the case of psychiatric disorder, I argued that, even if a psychological symptom *s_0_* is caused by an underlying neurological disturbance *c*, there could be further symptoms *s_0__+__n_* that arise from the phenomenal characters of *s_0_* and not as a direct consequence of *c.* To reveal the experiential processes involved in the relationship between *s_0_* and *s_0__+__n_* is the contribution of phenomenology.

In the case of psychological disturbances, I argued that CT fails to account for absolute cognitive primacy as it falls short in describing the development of schemas and the full scope of their nature. I then suggested that phenomenology is complementary to CT’s relative causative and limited constitutive cognitive primacy in that it allows for analyses of passive experiential processes which schematize experience directly (transition from *e_1_* to *e_2_*) or via the formation of certain beliefs (transition from *e_1_* to *b* to *e_2_*).

## Author Contributions

The author confirms being the sole contributor of this work and approved it for publication.

## Conflict of Interest Statement

The author declares that the research was conducted in the absence of any commercial or financial relationships that could be construed as a potential conflict of interest.
